# Experimental verification and molecular basis of active immunization against fungal pathogens in termites

**DOI:** 10.1038/srep15106

**Published:** 2015-10-13

**Authors:** Long Liu, Ganghua Li, Pengdong Sun, Chaoliang Lei, Qiuying Huang

**Affiliations:** 1 College of Plant Science and Technology, Huazhong Agricultural University, Wuhan 430070, Hubei, China

## Abstract

Termites are constantly exposed to many pathogens when they nest and forage in the field, so they employ various immune strategies to defend against pathogenic infections. Here, we demonstrate that the subterranean termite *Reticulitermes chinensis* employs active immunization to defend against the entomopathogen *Metarhizium anisopliae.* Our results showed that allogrooming frequency increased significantly between fungus-treated termites and their nestmates. Through active social contact, previously healthy nestmates only received small numbers of conidia from fungus-treated individuals. These nestmates experienced low-level fungal infections, resulting in low mortality and apparently improved antifungal defences. Moreover, infected nestmates promoted the activity of two antioxidant enzymes (SOD and CAT) and upregulated the expression of three immune genes (*phenoloxidase*, *transferrin*, and *termicin*). We found 20 differentially expressed proteins associated with active immunization in *R. chinensis* through iTRAQ proteomics, including 12 stress response proteins, six immune signalling proteins, and two immune effector molecules. Subsequently, two significantly upregulated (60S ribosomal protein L23 and isocitrate dehydrogenase) and three significantly downregulated (glutathione *S*-transferase D1, cuticle protein 19, and ubiquitin conjugating enzyme) candidate immune proteins were validated by MRM assays. These findings suggest that active immunization in termites may be regulated by different immune proteins.

To defend against pathogens, social insects have evolved a range of disease defences at the individual and colony level^1^,^2^,^3^,^4^,^5^. At the individual level, social insects fight off pathogens through innate physiological and biochemical systems such as cellular and humoral immunity^1^. At the colony level, social insects employ behavioural and social immunity to defend against pathogens, including allogrooming, trophallaxis, isolation, and cannibalism^2^,^6^,^7^,^8^,^9^. A recent study found active immunization in ants, which is characterized by social transfer of low-dose pathogens and active upregulation of nestmates’ immune systems during fungal infections^10^,^11^. Similarly, active immunization is also used to defend against diseases in humans, for example “variolation” is applied to protect humans against smallpox^12^. By comparison, passive immunization is characterized by social transfer of immune factors from infected individuals to naive nestmates, and it is also considered a type of social immunity in insect societies^13^. Also, the social transfer of immune elicitors can play a role in defending against pathogenic infections in social insects^14^,^15^, but the action mechanism of immune elicitors being transferred needs to be further studied^13^. Because passive immunization relies on transferring immune elicitors among nestmates, donors may face a direct physiological “trade-off” between individual immunity and social immunity^16^; for example, increased investment in personal immunity would cause decreased investment in social immunity^17^. Long-lived insect societies generally face high risks from the same pathogens during their lifespan^6^ and could thus benefit from the long-lasting protection of active immunization against pathogens rather than the temporary protection of passive immunization^10^. Termites often face serious pathogenic pressures when they nest and forage in the field^1^,^18^,^19^, so they have evolved various defence strategies to defend against pathogenic infections^3^,^4^,^5^,^8^,^9^. However, relatively little is known regarding active immunization against fungal pathogens in termites^20^.

Quantitative proteomics can identify and quantify the differentially expressed proteins in samples undergoing different treatments^21^. Recent successes in this field illustrate an increasingly important role of mass proteomics as an indispensable tool for identifying immune proteins to explain insect immune mechanisms^22^. For example, through proteome-wide correlation analyses, several proteins have been identified as highly predictive of social immunity behaviours in honey bees^23^. These biochemical correlations may reveal the molecular mechanisms underlying the social and innate immunity of honey bees^23^. Based on isobaric tagging for relative and absolute quantification (iTRAQ) analysis of *Tenebrio molitor* pupae resisting *Scleroderma guani* parasites, 41 proteins were identified and assigned to several functional categories including immunity, stress and detoxification^24^. Multiple reaction monitoring (MRM) with high-throughput confirmation has become a powerful tool for targeted proteomics^25^,^26^. Currently, iTRAQ discovery combined with subsequent MRM confirmation has been adopted to determine key protein biomarkers in diseases^25^,^26^. To date, it remains unclear which immune proteins take part in the regulation of active immunization in social insects. If iTRAQ proteomics and MRM assays are used together to look for immune proteins related to active immunization in social insects, we will achieve a better understanding of the molecular basis of active immunization in social insects.

The subterranean termites *Reticulitermes chinensis* Snyder are widely distributed throughout China, causing serious damage to structures and forests and bringing huge economic losses^27^,^28^. In this study, we used *R. chinensis* as the test organism to determine whether termites employ active immunization to defend against the entomopathogenic fungus *Metarhizium anisopliae* by employing multi-level methods, combing iTRAQ LC-MS/MS technology with MRM assays to find immune proteins involved in the active immunization of *R. chinensis*. Our results demonstrate that active immunization can be employed to defend against fungal pathogens in termites as originally hypothesized by Traniello *et al.* (2002)^8^, is a beneficial immune strategy for termite colonies, and may be regulated by different immune proteins.

## Results

### Termites increase allogrooming frequency after fungal infection

**Figure 1:**
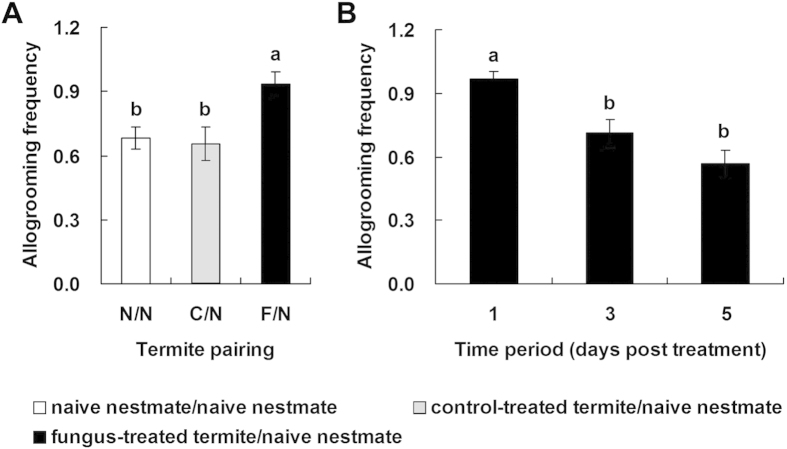
Observation of allogrooming behaviour for different treatment groups. (**A**) The allogrooming frequency of the different treatment groups. (**B**) Allogrooming frequency at different times between fungus-treated termites and naive nestmates over 5 d. Pairing groups include naive nestmates (N), control-treated termites (**C**), and fungus-treated termites (F). Error bars represent mean ± SEM. Different letters indicate significant differences (Tukey’s HSD test, *p* < 0.05).

The allogrooming frequency of the fungus-treated groups was significantly higher than that of the control-treated groups (Fig. 1A; *p* = 0.010) and the non-treated groups (*p* = 0.021), but there was no significant difference between the allogrooming frequency of the control-treated and non-treated groups (*p* = 0.948). Moreover, the allogrooming frequency on the first day was significantly higher than that on the third and fifth days in the fungus-treated groups (Fig. 1B; 1 d *vs.* 3 d: *p* = 0.011; 1 d *vs.* 5 d: *p* < 0.001), but there was no significant difference in allogrooming frequency between the third and fifth day (*p* = 0.191). There was no significant difference in the frequency of stomodeal trophallaxis between the three treatments (Supplementary Figure 1A; fungus-treated *vs.* control-treated: *p* = 0.811; fungus-treated *vs.* non-treated: *p* = 0.556; control-treated *vs.* non-treated: *p* = 0.238). Similarly, there was no significant difference in the frequency of stomodeal trophallaxis between the first, third, and fifth days (Supplementary Figure 1B; 1 d *vs.* 3 d: *p* = 0.911; 1 d *vs.* 5 d: *p* = 0.389; 3 d *vs.* 5 d: *p* = 0.629).

### Fungal detection and antifungal activity after social contact with a fungus-treated individual

The results of fungal detection showed that all fungus-treated individuals (9/9) and most of their nestmates (24/27) had conidia with blue fluorescence on their cuticles. Relatively high amounts of conidia were found in the legs (Supplementary Figure 2A and B) and abdomens (Supplementary Figure 2C) of fungus-treated individuals. Relatively low numbers of conidia were found in the legs (Supplementary Figure 2D and E) and abdomens (Supplementary Figure 2F) of nestmates. These results suggested that most of the nestmates received a low number of conidia from the fungus-treated individual through allogrooming.

**Figure 2:**
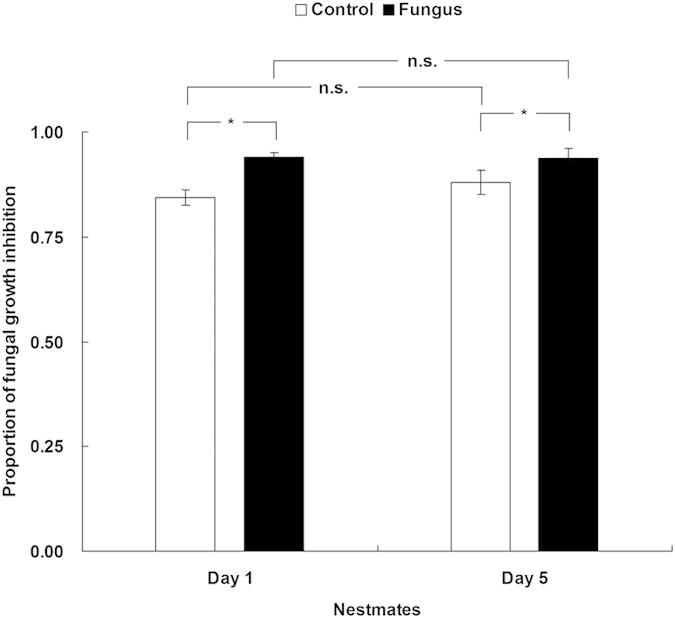
Antifungal activity of nestmates after contact with treated termites. Error bars represent mean ± SEM. Asterisks denote significant differences between nestmates of control-treated termites (white bars) and nestmates of fungus-treated termites (black bars) after 1 d and 5 d of social contact with the treated termites (*p < 0.05, paired *t*-test).

The antifungal activity in the nestmates of fungus-treated individuals was significantly higher than in the nestmates of control-treated individuals after 1 d of social interactions (Fig. 2; *t* = −4.523, *df* = 9, *p* = 0.001). This trend continued for the groups after 5 d of social interactions (Fig. 2; *t* = −2.331, *df* = 9, *p* = 0.045).

### Fungus transmission results in low-level infections and low mortality

**Figure 3:**
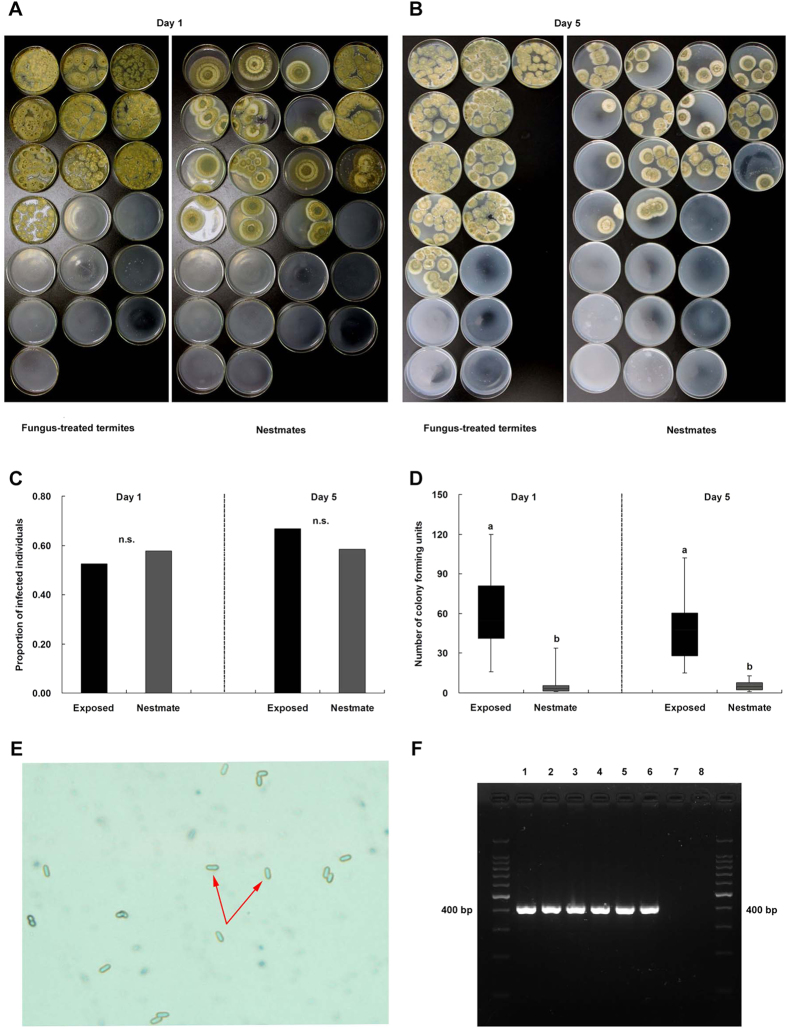
Nestmates experienced low-level *M. anisopliae* infections. (**A,B**) Growth of CFUs from fungus-treated termites and their nestmates after 1 d and 5 d of social contact. (**C,D**) Infection levels of fungus-treated termites (black bars) and their nestmates (grey bars) including the proportion of fungal growth and the number of CFUs after 1 d and 5 d of social contact (Mann-Whitney *U*-test, *p* < 0.05). (**E**) Identification of CFUs from conidia of nestmates of fungus-treated termites as *M. anisopliae* by morphological determination. (**F**) Identification of CFUs as *M. anisopliae* by PCR using primers specific for *M. anisopliae*, including PCR products of DNA from *M. anisopliae* (lanes 1 and 2, positive controls), from CFUs of fungus-treated termites (lanes 3 and 4), from CFUs of nestmates of fungus-treated termites (lanes 5 and 6) and from *Beauveria bassiana* (lanes 7 and 8, negative controls).

Fungal growth from the bodies of fungus-treated termites and nestmates is shown in Fig. 3A,B. After 1 d of social contact, we found that colony forming units (CFUs) grew from the bodies of 52.6% (10/19) of fungus-treated termites and 57.7% (15/26) of nestmates, and there was no significant difference between them (Fig. 3A,C; Fisher’s exact test, *p* = 0.569). The number of CFUs growing from nestmates was significantly lower than from fungus-treated individuals (Fig. 3A,D; Mann-Whitney *U*-test; *n*_*1*_ = 10, *n*_*2*_ = 15, *U* = 3.0, *p* < 0.001). After 5 d of social contact, we found that CFUs grew from the bodies of 66.67% (10/15) of fungus-treated termites and 58.33% (14/24) of nestmates, and there was no significant difference between them (Fig. 3B,C; Fisher’s exact test, *p* = 0.243). The number of CFUs growing from nestmates was significantly lower than from fungus-treated individuals (Fig. 3B,D; Mann-Whitney *U*-test; *n*_*1*_ = 10, *n*_*2*_ = 14, *U* = 0.0, *p* < 0.001). These results suggested that nestmates of fungus-treated termites were subjected to only low-level infections. All CFUs were confirmed as *M. anisopliae* by conidial morphology (Fig. 3E) and PCR products (Fig. 3F). Furthermore, we found that low-level infections occurring in nestmates resulted in low mortality (13%) of nestmates after 5 d of social contact with fungus-treated termites (50% mortality).

### Measurement of antifungal activity of antifungal substance transfer

**Figure 4:**
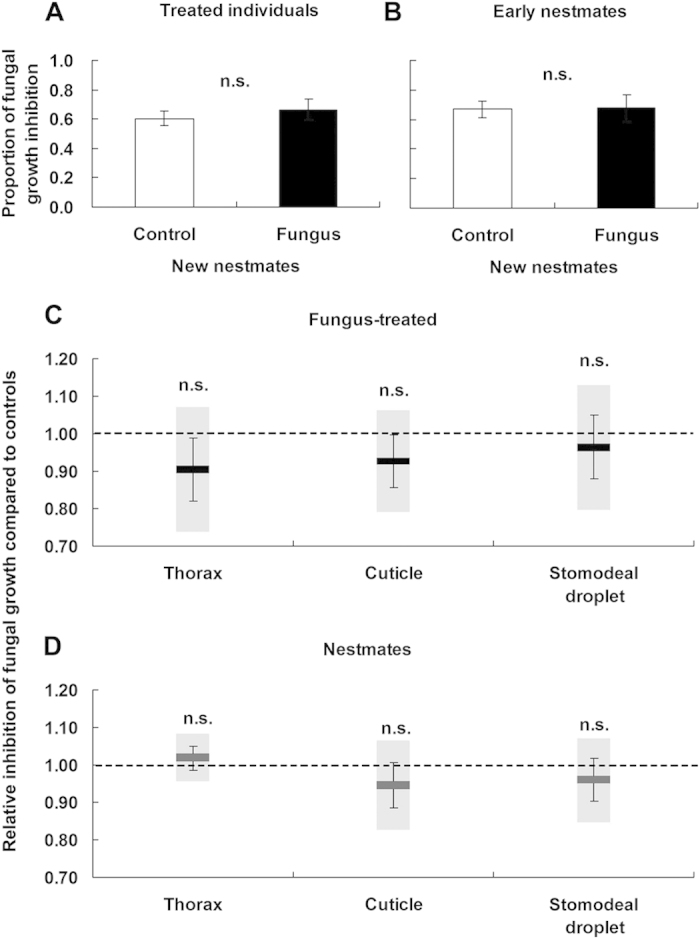
Antifungal activity assays of transferred antimicrobial substances. The antifungal activity of new nestmates of directly treated termites (**A**) and early nestmates (**B**) in a sham control assay (white bars) and under fungus treatment (black bars); Error bars represent mean ± SEM; n.s., not significant. Antifungal property of the exterior and interior of fungus-treated termites compared to control termites for directly treated termites (**C**) and their respective nestmates (**D**); Error boxplots represent mean ± SEM; n.s., not significant.

After 2 days of early social interactions, we separated the treated individual from its early nestmates, and then introduced five new nestmates to the treated individual and to its early nestmates for 5 days of maintenance. There was no significant difference in antifungal activity between the new nestmates of control-treated individuals and the new nestmates of fungus-treated individuals (Fig. 4A; *t* = −0.106, *df* = 7, *p* = 0.918). Similarly, there was no significant difference in antifungal activity between the new nestmates of early nestmates contacting control-treated individuals and the new nestmates of early nestmates contacting fungus-treated individuals (Fig. 4B; *t* = −0.744, *df* = 7, *p* = 0.481). Therefore, passive immunization had no impact on the antifungal activity of nestmates contacting a fungus-treated termite. After 5 days of social contact, we found no significant difference in the antifungal activity of the thorax, cuticle or stomodeal droplet between fungus-treated and control-treated termites (Fig. 4C; thorax: *t* = 1.133, *df* = 3, *p* = 0.300; cuticle: *t* = 1.059, *df* = 3, *p* = 0.367; stomodeal droplet: *t* = 0.422, *df* = 3, *p* = 0.701). Also, we found no significant difference in the antifungal activity of the thorax, cuticle or stomodeal droplet between the nestmates of fungus-treated individuals and the nestmates of control-treated individuals (Fig. 4D; thorax: *t* = −0.579, *df* = 3, *p* = 0.603; cuticle: *t* = 0.901, *df* = 3, *p* = 0.434; stomodeal droplet: *t* = 0.715, *df* = 3, *p* = 0.526). There was no presence of potentially transferable antimicrobial substances and no increase in the frequency of stomodeal trophallaxis (Supplementary Figure 1A and B), suggesting that passive immunization may not occur in *R. chinensis* during fungal infections.

### Activity of antioxidant enzymes and expression of immune genes in nestmates

**Figure 5:**
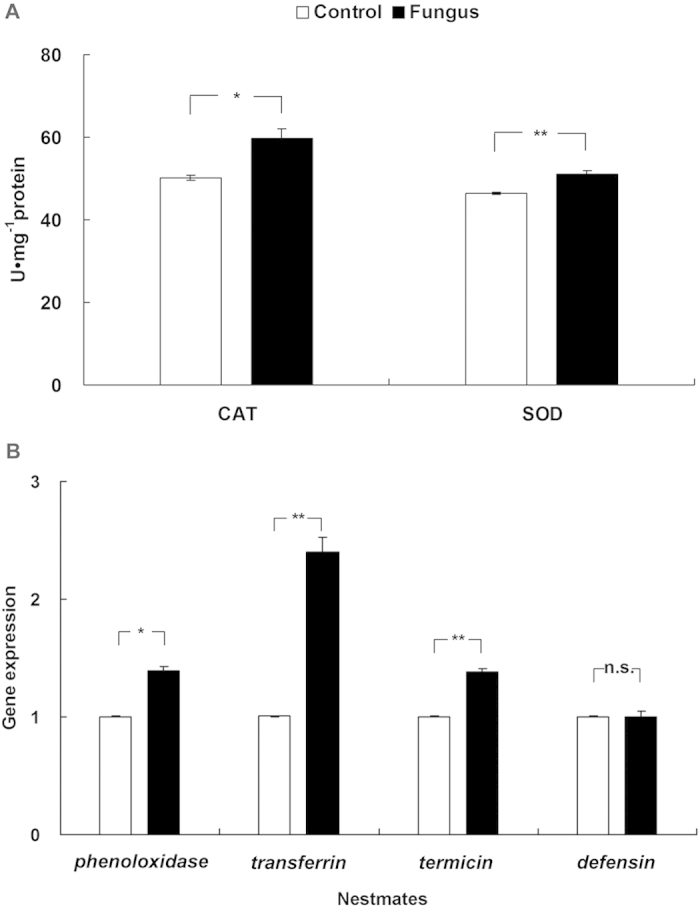
Activity of antioxidant enzymes and expression of immune genes in nestmates. (**A**) The activity of two antioxidant enzymes including catalase (CAT) and superoxide dismutase (SOD). (**B**) The expression of four immune genes including *phenoloxidase*, *transferrin*, *termicin*, and *defensin*. Error bars represent mean ± SEM. Asterisks denote significant differences between the nestmates of control-treated termites (white bars) and the nestmates of fungus-treated termites (black bars) after 5 d of social contact with treated termites. (*p < 0.05; **p < 0.01; n.s., not significant; paired *t*-test).

After 5 days of social contact, the activity of both superoxide dismutase (SOD) and catalase (CAT) in the nestmates of fungus-treated individuals was significantly higher than in the nestmates of control-treated individuals (Fig. 5A; SOD, *t* = 5.706, *df* = 4, *p* = 0.008; CAT, *t* = 3.942, *df* = 4, *p* = 0.017).

After 5 days of social contact, the expression levels of three immune genes in the nestmates of the fungus-treated individuals were significantly higher than in the nestmates of the control-treated individuals (Fig. 5B; *phenoloxidase*: *t* = −9.636, *df* = 2, *p* = 0.011; *transferrin*: *t* = −11.088, *df* = 2, *p* = 0.008; *termicin*: *t* = −3.819, *df* = 2, *p* = 0.005).

### Differentially expressed proteins associated with active immunization of *R. chinensis*

**Figure 6:**
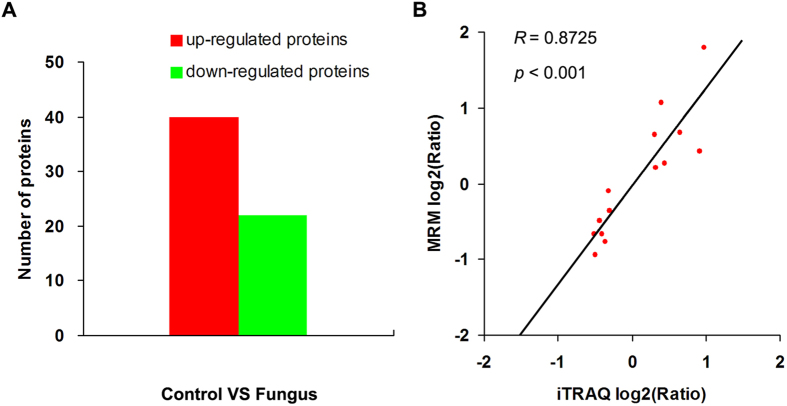
Differentially expressed proteins associated with active immunization in *R. chinensis*. (**A**) Number of differentially expressed proteins identified by iTRAQ (ratio >1.2 or <0.833, *p* < 0.05). Fungus represents nestmates of fungus-treated termites, and Control represents nestmates of control-treated termites. (**B)** The correlation between iTRAQ quantified log2 (protein ratio) and MRM quantified log2 (protein ratio) for the fourteen target proteins.

We identified 62 differentially expressed proteins (40 upregulated and 22 downregulated; ratio >1.2 or <0.833, *p* < 0.05) using iTRAQ proteomics (Fig. 6A). Among them, we found 20 candidate proteins associated with active immunization of *R. chinensis* (Supplementary Table 1). These proteins included 12 stress response proteins (one 60S ribosomal protein L23, one isocitrate dehydrogenase, three proteins involved in TcasGA2, two proteins involved in glutathione metabolism, one cuticle protein, one protein-disulfide isomerase, one cytochrome p450, and two chitin binding proteins), six immune signalling proteins (two small GTPases, two proteins of the ubiquitin-proteasome pathway, one transglutaminase, and one histone H3), and two immune effector proteins (two histones H1). The remaining 42 proteins were annotated to biosynthesis (3 proteins), metabolism (15 proteins), development (4 proteins), and other functions (20 proteins) (Supplementary Table 1).

### MRM validation for differentially expressed proteins from iTRAQ

MRM analysis succeeded in detecting 14 differentially expressed proteins from iTRAQ including 22 unique peptides in total. The transition information of the 14 target proteins can be found in Supplementary Table 2. The log ratios of the quantitative data of the 14 target proteins from MRM were significantly positively correlated with those from iTRAQ (Fig. 6B; *R* = 0.8725, *p* < 0.001). The four upregulated and the four downregulated proteins associated with active immunization from iTRAQ (Supplementary Table 1) have coincident expression with those from MRM (Table 1). Among them, the two significantly upregulated proteins (60S ribosomal protein L23 and isocitrate dehydrogenase) in iTRAQ (ratio > 1.2, *p* < 0.05) also showed significant upregulation in MRM (*p* < 0.05), and the three significantly downregulated proteins (glutathione *S*-transferase D1, cuticle protein 19, and ubiquitin conjugating enzyme) in iTRAQ (ratio < 0.833, *p* < 0.05) also showed significant downregulation in MRM (*p* < 0.05). In addition, the three upregulated proteins and the two downregulated proteins related to biosynthesis, metabolism, and development from iTRAQ (Supplementary Table 1) show coincident expression with those from MRM (Table 1). Among them, a significantly upregulated protein (transketolase-like protein 2) in iTRAQ also showed significant upregulation in MRM, and the two significantly downregulated proteins (hypothetical protein SINV_06138 and troponin i) in iTRAQ also showed significant downregulation in MRM.

## Discussion

Termites and other social insects employ individual innate immunity and social immunity to fight off pathogenic microorganisms throughout their life history^2^,^5^. Termite societies also use volatile chemicals to fumigate their nests to resist natural enemies^29^,^30^,^31^. Additionally, termites can use faecal pellets or antifungal proteins as nest material to defend against fungal infection^4^,^32^,^33^,^34^. Here, we demonstrate that termites employ active immunization similar to “variolation” in humans to defend against fungal pathogens. This immune strategy not only reduces the mortality risk of originally infected individuals but also allows nestmates to experience infections that are merely sublethal by actively upregulating their immune system as hypothesized by Traniello *et al.* (2002)^8^. Thus, active immunization is beneficial for termite colonies.

Active immunization can allow termite colonies to produce sufficient immune individuals to form a “protective wall”, which can prevent pathogens from transferring to reproductive castes (queens and kings) in termite colonies^35^. In comparison with passive immunization, active immunization likely results in individuals maintain their immunity longer due to active upregulation of their immune system^10^. Thus, active immunization at the group level likely results in the “protective wall” last longer than passive immunization. This ecological benefit of active immunization can explain why fungal pathogen outbreaks rarely occur in homeothermic termite colonies with high density under natural conditions^5^,^8^.

We found the significantly upregulated expression of three immune genes (*phenoloxidase*, *transferring*, and *termicin*) in the nestmates of fungus-exposed termites. *Phenoloxidase* plays a crucial role in the melanotic encapsulation of invaders, including entomopathogenic fungi^36^. *Transferrin* may isolate free iron ions in termite haemocoel and stop fungal growth due to a lack of ionic iron^37^. *Termicin* is an antimicrobial peptide (AMP) that plays a role in fighting off fungal pathogens in the innate immunity of insects^1^,^4^,^38^. We suspected that at least three upregulated immune genes may take part in active immunization in *R. chinensis*. Insects need to produce a large number of reactive oxygen species (ROS) to kill pathogens, but excessive ROS can result in damage to the organism^39^. Thus, insects need various antioxidant enzymes, such as SOD and CAT, to clear excessive ROS^40^,^41^. In our study, the significantly upregulated activity of both SOD and CAT provides evidence that variolated termites are responding to oxidative damage due to the transfer of sublethal dosages of the fungal pathogens.

Using MRM assays, we validated the two significantly upregulated proteins (isocitrate dehydrogenase and 60S ribosomal protein L23) and the two significantly downregulated proteins (glutathione *S*-transferase D1 and cuticle protein 19) among the 12 stress response proteins from iTRAQ. Isocitrate dehydrogenase is known as an important component in defence against oxidative stresses^42^,^43^. Ribosomal protein L23 is upregulated in honey bee larvae infected with chalkbrood fungus^44^. In our study, the two significantly upregulated proteins isocitrate dehydrogenase (Znev_13297) and 60S ribosomal protein L23 (Znev_11393) from iTRAQ were further confirmed by MRM, suggesting that they may play a role in the active immunization of *R. chinensis*. Additionally, glutathione *S*-transferase D1 (GSTD1) is an important detoxification enzyme, for example metabolizing the insecticide DDT in *Drosophila melanogaster*^45^. The cuticle is the first line of defence against pathogens in insects^46^. We found that the two significantly downregulated proteins GSTD1 (Znev_15569) and cuticle protein 19 (Znev_10404) from iTRAQ were further validated by MRM, suggesting that they may play a role in active immunization of *R. chinensis*.

We confirmed a significantly downregulated protein (ubiquitin conjugating enzyme) and a marginally significantly upregulated protein (GTPases Ras) among the six immune signalling proteins from iTRAQ. The ubiquitin-proteasome pathway plays an important role in the intracellular degradation of abnormal proteins that progressively accumulate under stress conditions^44^. The ubiquitin conjugating enzyme plays a key role in immune receptor signalsiling^47^. We found by iTRAQ and MRM that a ubiquitin conjugating enzyme (Znev_05594) of the ubiquitin-proteasome pathway was significantly downregulated, suggesting that this protein may play a role in active immunization in *R. chinensis*. A previous study found that JNK, small GTPases, and Eiger are required for prophenoloxidase release from crystal cells in *Drosophila*^48^, suggesting that small GTPases play a role in regulating the melanotic encapsulation of invaders. In our study, the upregulated GTPase Ras (Znev_14471) from iTRAQ was validated by MRM, indicating that this protein may be associated with active immunization in *R. chinensis*.

## Conclusions

Our results demonstrate that termites employ active immunization to defend against fungal pathogens. We found that previous naive nestmates acquire low-level fungal infections but not any antifungal substance through increased allogrooming frequency with fungus-exposed individuals. Moreover, these nestmates only experience low mortality with increased antifungal activity and upregulated activity of two antioxidant enzymes (SOD and CAT) and upregulated expression of three immune genes (*phenoloxidase*, *transferrin*, and *termicin*). These results indicate that active immunization is a beneficial immune strategy for termite colonies.

Our results suggest that active immunization in termites may be regulated by different immune proteins. Through iTRAQ proteomics, we found 20 differentially expressed proteins associated with the active immunization of *R. chinensis* through iTRAQ proteomics, including 12 stress response proteins, six immune signalling proteins, and two immune effector molecules. Furthermore, the two significantly upregulated candidate immune proteins (60S ribosomal protein L23 and isocitrate dehydrogenase) and the three significantly downregulated candidate immune proteins (glutathione *S*-transferase D1, cuticle protein 19, and ubiquitin conjugating enzyme) from iTRAQ proteomics were validated by MRM assays, suggesting that these five proteins may take part in the regulation of active immunization in *R. chinensis*. These findings provide new insight into the molecular basis of active immunization against fungal pathogens in termites.

## Methods

### Experimental termites

The subterranean termite *R. chinensis* was collected from Shizi, Yujia, Houshan, and the Nanwang hills of Wuhan city in the Hubei province of China. A total of 16 colonies of *R*. *chinensis* were used in this study (Supplementary Table 3). The termites were reared under laboratory conditions with a temperature of 25 °C ± 1 °C, 80% relative humidity and 24 h darkness.

### Fungal pathogen

Termite workers were treated with the entomopathogenic fungus *M. anisopliae* (strain IBCCM321.93). The fungus was cultivated on potato dextrose agar (PDA)^49^ for 2–4 weeks, and then it was collected with 0.1% Tween 80 to be made into a conidial suspension that could be stored at 4 °C for a maximum of 3–4 weeks^10^. Before each experiment, we measured conidial germination and found that all conidial suspensions had a germination rate of >95%. Termites were cold-immobilized and then were inoculated on their abdomens with a 0.35 μL droplet of the conidial suspension (10^7^ conidia/mL)^8^. They were used as fungus-treated individuals. Similarly, cold-immobilized termites were inoculated on their abdomens with a 0.35 μL droplet of conidia-free Tween 80 and were used as control-treated individuals^8^. After inoculation, all treated termites were refrigerated at 4 °C for an hour to lower their activity and to precipitate the conidia on their cuticle before being used in the following experiments^8^.

### Behavioural observation

We determined the frequency of allogrooming (mouth towards body; licking body surface and assuming the removal of pathogens) and frequency of stomodeal trophallaxis (mouth to mouth; assuming transferring of nutrients or antifungal substances)^13^,^50^,^51^. We determined the effect of membership in fungus-treated groups (fungus/naive), control-treated groups (Tween 80/naive), and non-treated groups (naive/naive) on the frequency of allogrooming and stomodeal trophallaxis (n = 10 replicates). Moreover, we determined the effect of different exposure times (1 d, 3 d, and 5 d) on the frequency of allogrooming and stomodeal trophallaxis in the fungus-treated group (n = 8 replicates). The above pairs were put together in a cell culture dish (3.5 cm in diameter, 1 cm high) with a piece of dampened filter paper and observed for 30 min after 3 min. In addition, we did not find any aggressive behaviour during observation.

### Experimental setup

We used a setup that included one fungus-treated termite marked with a black marking pen and five naive nestmates. Both the treated individuals and naive nestmates were kept in a Petri dish (9 cm diameter) with a piece of dampened filter paper as the food supply and were watered as required. After 1 d and 5 d of social contact, we measured the antifungal activity of the five nestmates (n = 10 replicates). To determine whether an antifungal substance was transferred, we maintained the treated individual and five nestmates together for 2 days, and then separated them. We then introduced five new nestmates separately to the treated individual and to one early nestmate in two separate experimental setups^10^. Because the conidia were bound to the termite cuticle two days after infection^52^,^53^, the conidia were no longer transferable, resulting in no transfer of conidia to the new nestmates. Thus, we could test whether antifungal substances could be transferred to the new nestmates. To examine whether the five new nestmates received the fungus, we assessed CFUs in the new nestmates (for details, see below). Finally, we measured the antifungal activity of the new nestmates after 5 d of interactions with treated individuals or early nestmates (n = 8 replicates). To further determine whether immune substances were transferred from the fungus-treated termites to the naive nestmates, the antifungal activity of the abdomen cuticles and thoraces (n = 4 replicates) and the trophallactic droplets (n = 4 replicates) of the treated termites and their respective nestmates were measured after 5 days of social contact. In control bioassays, we used 0.1% Tween 80 instead of conidia and performed the same operation as described above.

### Fungal detection

One fungus-treated termite and five naive termites were maintained for 2 d, and then we chose one directly exposed termite and randomly chose three nestmates per group (n = 9 groups; nine directly exposed termites and 27 nestmates in total), storing them at −40 °C. Before detection, solution A (10% KOH and 10% glycerine solution) and solution B (0.001% Calcofluor White M2Rstaining solution) (Sigma) were mixed equally. Samples were put in the mixed staining solution for 1 min, and then were washed with water, and subsequently were observed for fungal detection by using a fluorescence microscope (365 nm)^54^. Each sample was observed for a maximum of 30 min. In addition, we used the same method described above for fungal detection in 10 naive termites and did not find any structures resembling conidia labelled by the mixed staining solution.

### Antifungal activity assay

The antifungal activity of complete termites, dissected body parts (thorax and cuticle) and trophallactic droplets in different treatments was determined by the reduction of *M. anisopliae* fungal blastospores as measured by the absorbance in a microplate spectrophotometer^10^. For details of the procedures for antimicrobial substance extraction and measurement, see Supplementary Text 1.

### CFU determination

For CFU determination, we set up 20 groups each for 1 d and 5 d of social contact. Each group consisted of one directly exposed termite and five naive nestmates. At 1 d post-exposure, 19 exposed individuals and 26 nestmates were randomly picked up and then were frozen (−40 °C). Similarly, 15 exposed individuals and 24 nestmates were randomly picked up and then were frozen (−40 °C) at 5 d post-exposure. All the chosen samples were surface-sterilized in 75% ethanol for 30 s and 5% sodium hypochlorite for 3 min to destroy fungal material on the cuticle before dissection^55^. For each termite, the abdomen without cuticle was dissolved in 100 μL 50% glycerinum and then plated on selective medium containing 100 μg/mL chloramphenicol, 100 μg/mL streptomycin and 100 mg/mL kanamycin. After 16 d of cultivation for 1 d groups and 12 d of cultivation for 5 d groups at 25 °C ± 1 °C, the number of CFUs per selective medium was determined. All CFUs were identified as *M. anisopliae* by conidial morphology and specific primers (Supplementary Text 1).

For negative controls, we performed the same protocols described above to determine whether the fungus grew from 10 unexposed termites or 10 termites that were exposed to *M. anisopliae* and then were surface-sterilised after 3 hours. We did not find fungal growth from these 20 termites. To determine whether new nestmates of fungus-treated individuals or their early nestmates were infected by *M. anisopliae*, we also used the method described above. The results showed that none of the new nestmates displayed fungal growth (n = 8 replicates each), thus confirming that the new nestmates did not receive conidia.

### Activity of antioxidant enzymes and expression of immune genes

Activity of two antioxidant enzymes were analysed in pools of 15 nestmates for each of the five colonies. To determine the effect of active immunization on nestmates, we chose SOD^40^ and CAT^41^. Details of the antioxidant enzyme measurement methods are described in Supplementary Text 1. Nestmates of treated termites were analysed by qPCR for the expression of four immune genes including *phenoloxidase* (PO^36^), *transferrin*^37^, *termicin*^1^,^38^, and *defensin*^10^. Details of the methods of RNA extraction, primer design, PCR application and qPCR are described in Supplementary Text 1.

### Quantitative iTRAQ analysis and MRM assays

One fungus-treated (or control-treated) termite and five nestmates were maintained together for 5 d. The nestmates of fungus-treated termites were regarded as the treatment sample, and the nestmates of control-treated termites were regarded as the control sample. There were three replicates for both the treatment (Fungus 1, Fungus 2, and Fungus 3) and control (Control 1, Control 2, and Control 3) samples. Each sample consisted of 100 mg of the above nestmates. To look for differentially expressed proteins associated with active immunization of *R. chinensis*, we performed quantitative iTRAQ LC−MS/MS proteomic analysis. Details of iTRAQ analysis are given in Supplementary Text 1. Subsequently, we used MRM assays to validate the differentially expressed proteins from iTRAQ. Details of the MRM analysis are described in Supplementary Text 1.

### Statistical analyses

All of the data were analysed with IBM SPSS (Statistical Package for the Social Sciences) Statistics 19.0 software. The antifungal activity, enzymatic activity and gene expression of nestmates contacting treated individuals were analysed using paired *t*-tests. Behavioural observations were analysed using one-way ANOVAs, and significant differences were analysed using Tukey’s HSD test. The antifungal activities of the thorax, cuticle and trophallactic droplet were analysed using *t*-tests. The proportion of infected individuals (at least a single CFU in each selective medium) between directly exposed termites and their nestmates was analysed by the Fisher exact test. The number of CFUs from dissected body contents between directly exposed termites and their nestmates was analysed by Mann-Whitney *U*-test^10^.

## Additional Information

**How to cite this article**: Liu, L. *et al.* Experimental verification and molecular basis of active immunization against fungal pathogens in termites. *Sci. Rep.* **5**, 15106; doi: 10.1038/srep15106 (2015).

## Supplementary Material

Supplementary Information

## Figures and Tables

**Table 1:** MRM validation of differentially expressed proteins associated with active immunization in *R. chinensis.*

Function	Protein name	Unigene ID	Peptide sequence	Fold change (Mean)	*P*-value
Stress response proteins	60S ribosomal protein L23	Znev_11393	DYDALDVANK	1.57	0.01
	isocitrate dehydrogenase	Znev_13297	VTIIPGDGIGPEISAAVQK	1.65	0.04
		Znev_13297	SLEGYETLYDNVDVVTIR	1.22	0.29
		Znev_13297	ENTEGEYSGIEHEIVDGVVQSIK	1.94	0.02
	glutathione *S*-transferase D1	Znev_15569	LYFDIGTLYQR	0.59	0.00
	cuticle protein 19	Znev_10404	AAPAVDYYAYPK	0.61	0.05
		Znev_10404	GEYSLVEPDGTVR	0.44	0.01
	protein-disulfide isomerase	Znev_03262	VLVSSNFDEVAFNK	1.34	0.14
Immune signaling molecules	GTPase Ras	Znev_14471	SFEDIGGYR	2.10	0.06
	ubiquitin conjugating enzyme	Znev_05594	TDQVIQALVALVNDPEPEHPLR	0.57	0.00
		Znev_05594	ADLAEEYLK	0.69	0.00
	26S proteasome	Znev_05722	IVAFVGSPVETEEK	0.94	0.45
Biosynthesis	hypothetical protein SINV_06138	Znev_09984	LFIGGLDYR	0.67	0.00
		Znev_09984	GFGFITYSR	0.92	0.50
		Znev_09984	GFGFVEFEDYDPVDK	0.54	0.01
	copa protein	Znev_06668	LVGQSIIAYLQQK	1.03	0.68
		Znev_06668	LSFLYLITGNLDK	0.52	0.01
Metabolism	transketolase-like protein 2	Znev_11074	SIPGSTVFYPSDAVSTER	3.46	0.00
	phosphoglycerate mutase 1	Znev_02731	FDIAHTSVLTR	1.21	0.61
Development	troponin i	Znev_15251	FDLEYAVK	0.61	0.00
		Znev_15251	DFEISDLNAQVNDLR	0.65	0.02
	PREDICTED: annexin-B9-like	Znev_05200	EFAGSLEDGYLSIVK	1.16	0.40
