# Supplementary material for: Experimental verification and molecular basis of active immunization against fungal pathogens in termites
Source: Sci Rep. 2015 Oct 13;5:15106. doi: 10.1038/srep15106 (PMC4602225; doi:10.1038/srep15106)
Supplement: Supplementary Information [file srep15106-s1.pdf]

SUPPLEMENTARY INFORMATION

**Experimental verification and molecular basis of active immunization against fungal pathogens in termites**

Long Liu [#], Ganghua Li [#], Pengdong Sun, Chaoliang Lei, Qiuying Huang [*]
College of Plant Science and Technology, Huazhong Agricultural University, Wuhan 430070, Hubei, China

[#] These authors contributed equally to this work

* Correspondence: Dr. Qiuying Huang
No.1, Shizishan Street, Hongshan District, Wuhan 430070, China
E-mail: qyhuang2006@mail.hzau.edu.cn Tel: 86-27-87287207

**Supplementary Figure**

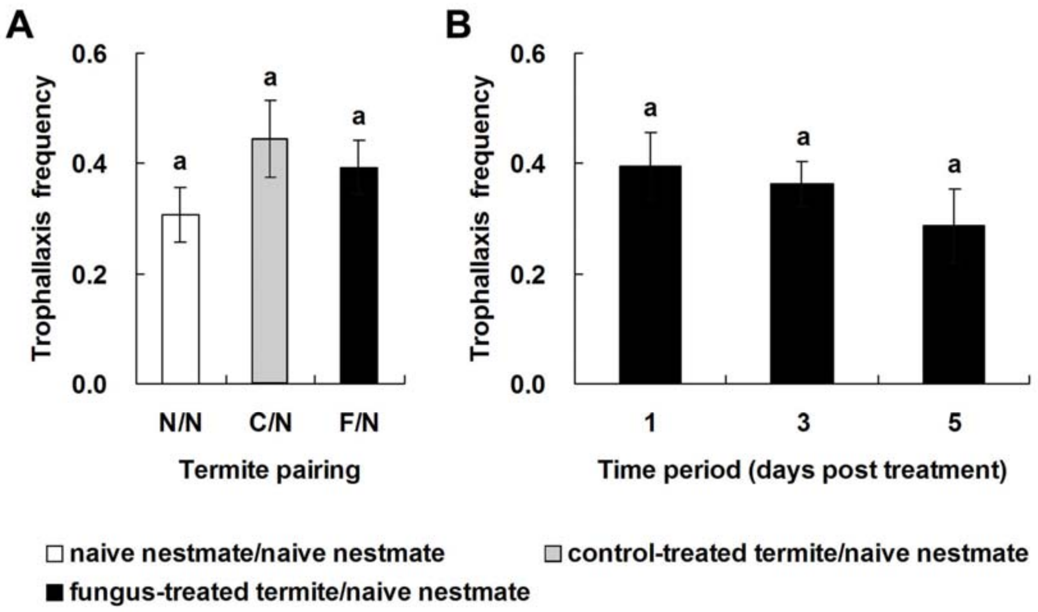


**Supplementary Figure 1. Observation of stomodeal trophallaxis for different treatment groups.**

(A) The frequency of stomodeal trophallaxis of different treated groups. (B) The frequency of stomodeal trophallaxis at different times between fungus-treated termites and naive nestmates over 5 d. Pairing groups include naive nestmates (N), control-treated termites (C) and fungus-treated termites (F). Error bars represent mean ± SEM. Different letters indicate significant differences (Tukey's HSD test, $p < 0.05$).

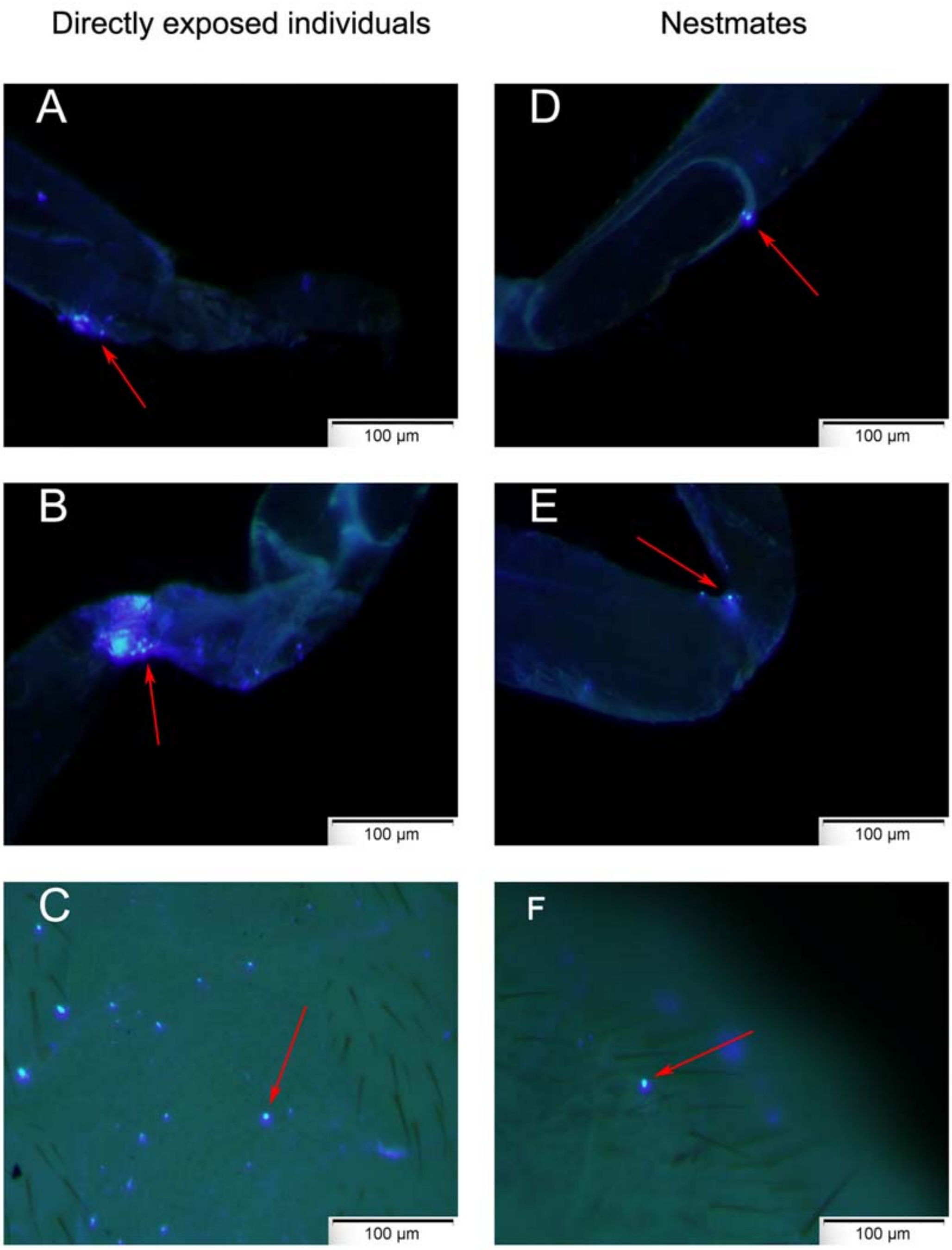


**Supplementary Figure 2. Detection of conidia on fungus-treated termites and their nestmates.**

Conidia were found on the legs (A), joints of the legs (B) and abdominal cuticle (C) of the fungus-treated termites. In comparison, relatively low amounts of conidia were found on the legs (D), joints of the legs (E) and abdominal cuticle (F) of their nestmates.

**Supplementary Table 1.** Identification of differentially expressed proteins in *R. chinensis* infected by *M. anisopliae* using iTRAQ proteomics.

| Function | Protein name | Unigene ID | Accession No. | Score | E-value | Fold change (Mean) |
|---|---|---|---|---|---|---|
| Stress response | 60S ribosomal protein L23 | Znev_11393 | XP_001942614.1 | 126 | 4.00E-66 | 1.24 |
| | isocitrate dehydrogenase | Znev_13297 | EEZ99082.1 | 952 | 0 | 1.57 |
| | glutathione *S*-transferase D1 | Znev_15569 | AFK49803.1 | 288 | 4.00E-109 | 0.78 |
| | cuticle protein 19 | Znev_10404 | P45583.1 | 445 | 2.00E-24 | 0.71 |
| | protein-disulfide isomerase | Znev_03262 | XP_975184.2 | 276 | 0 | 1.89 |
| | glutathione *S*-transferase | Znev_00817 | AFZ78680.1 | 190 | 4.00E-111 | 2.22 |
| | cytochrome P450 | Znev_14802 | BAD84176.1 | 223 | 1.00E-169 | 1.91 |
| | chitinase | Znev_05886 | XP_003436306.1 | 54 | 5.00E-105 | 0.71 |
| | chitin metabolic | Znev_18457 | XP_001945816.1 | 57 | 5.00E-46 | 0.66 |
| | hypothetical protein TcasGA2_TC000103 | Znev_08435 | EEZ99522.1 | 155 | 0 | 0.79 |
| | hypothetical protein TcasGA2_TC013127 | Znev_05812 | EFA03207.1 | 49 | 4.00E-12 | 1.65 |
| | hypothetical protein TcasGA2_TC015417 | Znev_14079 | EFA05262.1 | 240 | 6.00E-129 | 0.77 |
| Immune signaling | GTPase Ras | Znev_14471 | XP_307965.4 | 111 | 3.00E-84 | 1.32 |
| | ubiquitin conjugating enzyme | Znev_05594 | XP_001518397.2 | 170 | 2.00E-31 | 0.70 |
| | 26S proteasome | Znev_05722 | DAA34352.1 | 366 | 2.00E-68 | 0.80 |
| | Ras-related protein | Znev_00355 | XP_002024575.1 | 131 | 4.00E-53 | 1.29 |
| | transglutaminase | Znev_13185 | P52183.1 | 112 | 0 | 1.89 |
| | histone H3 | Znev_04751 | NP_002098.1 | 121 | 2.00E-72 | 5.84 |
| Immune effector | histone H1-II-1 | Znev_12492 | XP_002422630.1 | 189 | 4.00E-19 | 4.56 |
| | histone H1-II | Znev_12491 | EFA13427.1 | 167 | 3.00E-25 | 2.44 |
| Biosynthesis | small nuclear ribonucleoprotein F | Znev_18326 | EFN72155.1 | 65 | 2.00E-33 | 4.37 |
| | hypothetical protein SINV_06138 | Znev_09984 | EFZ21040.1 | 351 | 6.00E-93 | 0.74 |
| | copa protein | Znev_06668 | AAI50356.1 | 83 | 5.00E-42 | 0.81 |

| | | | | | | |
|---|---|---|---|---|---|---|
| Metabolism | ATP synthase oligomycin sensitivity conferral protein | Znev_00096 | ABD98749.1 | 61 | 7.00E-75 | 1.79 |
| | transketolase-like protein 2 | Znev_11074 | EFN73921.1 | 240 | 0 | 1.96 |
| | phosphoglycerate mutase 1 | Znev_02731 | EFN64748.1 | 102 | 5.00E-120 | 1.35 |
| | serine/threonine-protein phosphatase alpha-1 isoform | Znev_03910 | EFN86649.1 | 145 | 7.00E-158 | 0.68 |
| | PREDICTED: tubulin alpha-1C chain-like | Znev_15255 | XP_003826076.1 | 2192 | 4.00E-56 | 0.62 |
| | hypothetical protein DAPPUDRAFT_305931 | Znev_15191 | EFX63892.1 | 86 | 3.00E-93 | 1.93 |
| | PREDICTED: 60S ribosomal protein L31-like | Znev_12203 | XP_003491807.1 | 62 | 8.00E-50 | 1.43 |
| | ribosomal protein | Znev_03722 | CAB46822.1 | 96 | 2.00E-48 | 1.37 |
| | hypothetical protein AND_02423 | Znev_01471 | EFR28979.1 | 107 | 2.00E-28 | 1.53 |
| | proteasome subunit beta 7 | Znev_02538 | EHJ64129.1 | 52 | 3.00E-104 | 1.77 |
| | PREDICTED: eukaryotic translation initiation factor 2 subunit 1-like | Znev_03136 | XP_003691549.1 | 108 | 4.00E-153 | 1.74 |
| | hypothetical protein SINV_10530 | Znev_03341 | EFZ16690.1 | 132 | 3.00E-41 | 1.77 |
| | ribosomal protein S12 | Znev_05370 | AAM33784.1 | 60 | 5.00E-68 | 0.55 |
| | SUI protein | Znev_01101 | ADJ68166.1 | 54 | 4.00E-29 | 0.71 |
| | ribosomal protein L36e | Znev_10183 | CAR94544.1 | 59 | 4.00E-55 | 0.72 |
| Development | hypothetical protein SINV_02609 | Znev_01719 | EFZ11601.1 | 199 | 0 | 1.71 |
| | troponin i | Znev_15251 | XP_001661109.1 | 366 | 3.00E-39 | 0.75 |
| | PREDICTED: annexin-B9-like | Znev_05200 | XP_003693405.1 | 283 | 8.00E-121 | 1.25 |
| | aldo-keto reductase, partial | Znev_12732 | AEV89779.1 | 127 | 2.00E-91 | 1.40 |
| Other functions | group II PLP decarboxylase | Znev_05056 | XP_002433673.1 | 115 | 3.00E-19 | 0.66 |
| | hypothetical protein SINV_15420 | Znev_07932 | EFZ09502.1 | 198 | 7.00E-23 | 1.33 |
| | PREDICTED: prostaglandin reductase 1-like | Znev_13724 | XP_001603755.1 | 70 | 1.00E-107 | 3.57 |
| | n/a | Znev_09000 | n/a | 169 | n/a | 1.38 |
| | hypothetical protein SINV_11708 | Znev_03344 | EFZ21891.1 | 39 | 1.00E-58 | 1.84 |
| | AGAP003540-PA | Znev_02356 | XP_562377.4 | 32 | 2.00E-09 | 6.46 |
| | MBD2-interacting zinc finger, isoform CRA_a | Znev_06037 | EAW67460.1 | 26 | 4.00E-65 | 1.37 |

| | | | | | |
|---|---|---|---|---|---|
| PREDICTED: uncharacterized protein LOC755912, partial | Znev_04048 | XP_001192548.2 | 114 | 2.00E-07 | 1.83 |
| PREDICTED: similar to AGAP007325-PA isoform 5 | Znev_07036 | XP_976127.1 | 144 | 5.00E-41 | 1.70 |
| PREDICTED: LOW QUALITY PROTEIN: protein CLP1 homolog | Znev_01522 | XP_003401895.1 | 33 | 0 | 1.58 |
| PREDICTED: protein ETHE1, mitochondrial-like | Znev_04335 | XP_003481924.1 | 45 | 4.00E-14 | 2.31 |
| D-aspartate oxidase | Znev_06652 | EGI66218.1 | 25 | 2.00E-11 | 2.03 |
| haloacid dehalogenase-like hydrolase domain-containing protein 2 | Znev_12098 | EFN61412.1 | 124 | 4.00E-85 | 1.57 |
| hypothetical protein DAPPUDRAFT_18429 | Znev_09951 | EFX73538.1 | 33 | 6.00E-18 | 2.21 |
| GF22955 | Znev_12980 | XP_001964611.1 | 122 | 4.00E-38 | 1.25 |
| PREDICTED: sulfide:quinone oxidoreductase, mitochondrial-like | Znev_01082 | XP_003730634.1 | 159 | 1.00E-18 | 0.76 |
| PREDICTED: hypothetical protein LOC100575643 | Znev_16651 | XP_003247465.1 | 102 | 7.00E-06 | 0.72 |
| n/a | Znev_02478 | n/a | 77 | n/a | 0.57 |
| PREDICTED: hypothetical protein LOC409010 | Znev_08111 | XP_392539.3 | 58 | 9.00E-15 | 0.43 |
| hypothetical protein AND_20271 | Znev_03253 | EFR20331.1 | 81 | 1.00E-131 | 0.77 |

**Supplementary Table 2.** Sequences and transitions of 14 target proteins detected by MRM.

| Protein name | Unigene ID | Peptide sequence | Quadrupole 1 | Quadrupole 3 | Collision energy | Retention time (min) |
|---|---|---|---|---|---|---|
| 60S ribosomal protein L23 | Znev_11393 | DYDALDVANK | 562.266935 | 845.436323 | 29.1 | 15.73 |
| | | | 562.266935 | 546.288202 | 29.1 | 15.73 |
| | | | 562.266935 | 431.261259 | 29.1 | 15.73 |
| isocitrate dehydrogenase | Znev_13297 | VTIIPGDGIGPEISAAVQK | 933.022565 | 999.546936 | 42.4 | 22.91 |
| | | | 933.022565 | 942.525472 | 42.4 | 22.91 |
| | | | 933.022565 | 603.346051 | 42.4 | 22.91 |
| | | SLEGYETLYDNVDVVTIR | 696.016148 | 702.414465 | 35.5 | 23.77 |
| | | | 696.016148 | 587.387522 | 35.5 | 23.77 |
| | | | 696.016148 | 488.319108 | 35.5 | 23.77 |
| | | ENTEGEYSGIEHEIVDGVVQSIK | 844.741523 | 944.541122 | 43.5 | 25.09 |
| | | | 844.741523 | 730.445765 | 43.5 | 25.09 |
| | | | 844.741523 | 475.287474 | 43.5 | 25.09 |
| glutathione *S*-transferase D1 | Znev_15569 | LYFDIGTLYQR | 694.864077 | 965.505071 | 33.9 | 24.34 |
| | | | 694.864077 | 850.478128 | 33.9 | 24.34 |
| | | | 694.864077 | 737.394064 | 33.9 | 24.34 |
| cuticle protein 19 | Znev_10404 | AAPAVDYYAYPK | 664.829703 | 1018.488024 | 32.8 | 16.93 |
| | | | 664.829703 | 919.41961 | 32.8 | 16.93 |
| | | | 664.829703 | 804.392667 | 32.8 | 16.93 |
| | | GEYSLVEPDGTVR | 711.348988 | 773.378808 | 34.5 | 17.22 |
| | | | 711.348988 | 644.336215 | 34.5 | 17.22 |
| | | | 711.348988 | 432.256508 | 34.5 | 17.22 |
| protein-disulfide isomerase | Znev_03262 | VLVSSNFDEVAFNK | 784.901388 | 969.467623 | 37.1 | 21.13 |
| | | | 784.901388 | 822.399209 | 37.1 | 21.13 |

| | | | | | | |
|---|---|---|---|---|---|---|
| | | | 784.901388 | 479.261259 | 37.1 | 21.13 |
| GTPase Ras | Znev_14471 | SFEDIGGYR | 522.243263 | 809.378808 | 27.7 | 16.65 |
| | | | 522.243263 | 680.336215 | 27.7 | 16.65 |
| | | | 522.243263 | 565.309272 | 27.7 | 16.65 |
| ubiquitin conjugating enzyme | Znev_05594 | TDQVIQALVALVNDPEPEHPLR | 818.771089 | 974.505406 | 42.1 | 27.21 |
| | | | 818.771089 | 748.410049 | 42.1 | 27.21 |
| | | | 818.771089 | 522.314692 | 42.1 | 27.21 |
| | | ADLAEEYLK | 526.268947 | 865.46656 | 27.8 | 18.60 |
| | | | 526.268947 | 752.382496 | 27.8 | 18.60 |
| | | | 526.268947 | 552.302789 | 27.8 | 18.60 |
| 26S proteasome | Znev_05722 | IVAFVGSPVETEEK | 752.898314 | 975.462932 | 36.0 | 19.00 |
| | | | 752.898314 | 831.409439 | 36.0 | 19.00 |
| | | | 752.898314 | 506.245669 | 36.0 | 19.00 |
| hypothetical protein SINV_06138 | Znev_09984 | LFIGGLDYR | 527.290016 | 940.488693 | 27.8 | 21.59 |
| | | | 527.290016 | 793.420279 | 27.8 | 21.59 |
| | | | 527.290016 | 680.336215 | 27.8 | 21.59 |
| | | GFGFITYSR | 524.266541 | 843.435929 | 27.7 | 21.07 |
| | | | 524.266541 | 639.346051 | 27.7 | 21.07 |
| | | | 524.266541 | 526.261987 | 27.7 | 21.07 |
| | | GFGFVEFEDYDPVDK | 882.393592 | 980.420732 | 40.6 | 24.06 |
| | | | 882.393592 | 851.378139 | 40.6 | 24.06 |
| | | | 882.393592 | 458.260925 | 40.6 | 24.06 |
| copa protein | Znev_06668 | LVGQSIIAYLQQK | 730.927208 | 1063.614622 | 35.2 | 20.21 |
| | | | 730.927208 | 863.498529 | 35.2 | 20.21 |
| | | | 730.927208 | 679.377351 | 35.2 | 20.21 |
| | | LSFLYLITGNLDK | 748.921592 | 873.504009 | 35.8 | 28.42 |

| | | | | | | |
|---|---|---|---|---|---|---|
| | | | 748.921592 | 760.419945 | 35.8 | 28.42 |
| | | | 748.921592 | 546.288202 | 35.8 | 28.42 |
| transketolase-like protein 2 | Znev_11074 | SIPGSTVFYPSDAVSTER | 638.314417 | 961.458515 | 32.3 | 20.38 |
| | | | 638.314417 | 864.405751 | 32.3 | 20.38 |
| | | | 638.314417 | 662.34678 | 32.3 | 20.38 |
| phosphoglycerate mutase 1 | Znev_02731 | FDIAHTSVLTR | 420.562938 | 813.457727 | 20.5 | 17.74 |
| | | | 420.562938 | 676.398815 | 20.5 | 17.74 |
| | | | 420.562938 | 575.351137 | 20.5 | 17.74 |
| troponin i | Znev_15251 | FDLEYAVK | 492.755475 | 837.43526 | 26.6 | 19.69 |
| | | | 492.755475 | 722.408317 | 26.6 | 19.69 |
| | | | 492.755475 | 480.28166 | 26.6 | 19.69 |
| | | DFEISDLNAQVNDLR | 874.926122 | 929.479919 | 40.4 | 23.08 |
| | | | 874.926122 | 744.399878 | 40.4 | 23.08 |
| | | | 874.926122 | 517.272886 | 40.4 | 23.08 |
| PREDICTED: annexin-B9-like | Znev_05200 | EFAGSLEDGYLSIVK | 814.414328 | 1023.535703 | 38.2 | 23.65 |
| | | | 814.414328 | 894.49311 | 38.2 | 23.65 |
| | | | 814.414328 | 559.381374 | 38.2 | 23.65 |

**Supplementary Table 3.** The distribution of the 16 colonies of *R. chinensis* for each experiment.

| Experiments | Replicates | Colonies |
|---|---|---|
| Behavioural observation | 10 | Colony number 1, 3, 4, 6, 7, 10, 11, 12, 13, 16 |
| Fungal detection | 9 | Colony number 1, 2, 3, 7, 10, 11, 12, 13, 16 |
| Antifungal activity | 10 | Colony number 1, 3, 4, 6, 7, 10, 11, 12, 13, 16 |
| CFUs observation | 5 | Colony number 5, 9, 10, 11, 15 |
| Antifungal substances transfer (new nestmates) | 8 | Colony number 2, 3, 7, 10, 11, 12, 15, 16 |
| Antifungal substances transfer (body parts) | 4 | Colony number 2, 3, 7, 12 |
| Antioxidant enzymes | 5 | Colony number 3, 7, 12, 13, 16 |
| Gene expression | 3 | Colony number 2, 7, 10 |
| iTRAQ proteomics | 3 | Colony number 4, 8, 14 |
| MRM assays | 3 | Colony number 4, 8, 14 |

**Supplementary Text 1.** Experimental protocols.

**Antifungal activity assay**

For each replicate per treated type consisting of a pool of five individuals, we crushed five termites in centrifuge tubes with liquid nitrogen and then used phosphate buffered saline (PBS) to dissolve them in a proportion of 1 mg of body weight to 5 μL of PBS. Then, the homogenates were centrifuged at 6000 x g for five minutes at 4 °C and then 20 μL of the extract supernatants were centrifuged at 6000 x g for five minutes at 4 °C again. Then, 10 μL of the supernatants were extracted and stored at -80 °C until antifungal activity assay. For the antifungal activity of the two body parts (abdomen cuticle and thorax), we froze and dissected 20 fungus-treated and 20 control-treated termites. Each treated type consisting of a pool of abdomen cuticles and thoraces of five termites were crushed in liquid nitrogen in each replicate, then were dissolved in 50 μL of PBS and performed as the methods described above. For the antifungal activity of stomodeal droplet, we obtained the stomodeal droplets of 20 fungus-treated individuals and 20 control-treated individuals. Because we only retrieved 0.1 μL regurgitate from one termite with capillary, we pooled stomodeal droplets of five termites in each replicate and dissolved stomodeal droplets in 1.5 μL of PBS. The supernatant of abdomen cuticles and thoraces, and the stomodeal droplet were stored at -80 °C until antifungal activity assay. When measuring antifungal activity, we used 96-well microplates with 50 μL potato dextrose (PD), 2 μL blastospores ($10^6$ spores/mL), 2 μL supernatant or stomodeal droplets per well. Additionally, we used 50 μL PD, 2 μL the blastospores, 2 μL PBS per well for spores-growth control, and 50 μL PD, 4 μL PBS per well for standards. After 24 hours of cultivation in constant temperature shaker (200 rpm; 25 °C ± 1 °C), the absorbance of each well was measured by the microplate spectrophotometer (wavelength: 600 nm).

**Confirmation of identity of CFUs as *M. anisopliae* by PCR.**

We extracted DNA of *M. anisopliae* (strain IBCCM321.93) as positive controls and that of *Beauveria bassiana* (strain GIM3.428) as negative controls directly from our fungal culture. We also extracted DNA of CFUs from fungus-treated termites and their nestmates to determine whether the CFUs from dissected body contents of the termites were truly *M. anisopliae*.

DNA was extracted by the method of CTAB. The fungal material was scraped lightly from the Petri Dish, and crushed in liquid nitrogen for 30 seconds, and then heated at 65 °C for 30 second. The procedure was repeated for three times. The fungal material was then resuspended with 1 mL CTAB buffer (2% CTAB, 0.75 M NaCl, 50 mM Tris/HCl pH 8.0, 10 mM EDTA) and then add 10 μL Proteinase K (TaKaRa) to the suspension. Each suspension was incubated for 1 hour at 55 °C and mixed by vortexing for 5 seconds every 10 minutes. We added 1 mL chloroform/isoamyl alcohol (24:1) and mixed. The mixture was centrifuged at 12 000×g for 10 minutes. The upper phase (contain DNA) was removed to a new 1.5 mL tube. The DNA was extracted by chloroform/isoamyl alcohol (24:1) for two times by the method described above to remove the protein in DNA. The upper phase (about 300 μL) was incubated at -20 °C for 30 minutes with 600 μL ethyl alcohol and 30μL NaAc (3 M). Then the mixture was centrifuged at 12 000 ×g for 15 minutes to collect the DNA. DNA pellets were washed by 1 mL 75% ethyl alcohol. After centrifuged at 12 000 ×g for 10 minutes, the solution was abandoned. DNA pellets were dried at room temperature for 15 minutes and then dissolved by 50μL $ddH_2O$.

The universal primer: ITS1 (5'-TCCGTAGGTGAACCTGCGG-3') and ITS4 (5'-TCCTCCGCTTATTGATATGC-3') were used to amplify the sequence of *M. anisopliae*. The protocol of PCR action contains 12.8 μL of $ddH_2O$, 0.4 μL of ITS1 (10 μM), 0.4 μL of ITS4 (10 μM), 1 μL of DNA template, 3.2 μL of dNTP Mixture (2.5 mM each), 2 μL of 10×LA Taq Buffer II ($Mg^{2+}$ Plus) and 0.2 μL of TaKaRa LA Taq (5 U/μL). PCR reactions were performed in a thermocycler (MyCycler™ Thermal Cycler, Bio-Rad) with the following amplification conditions: 4 minutes denaturation of 95 °C, followed by 35 cycles of 95 °C for 30 seconds, 55 °C for 30 seconds and 72 °C for 1 minute, and final extension at 72 °C for 7 minutes. The PCR products were purified by Wizard® SV Gel and PCR Clean-Up System, and the sequence of gel extraction products was confirmed by sequencing.

The specific primer for *M. anisopliae* (forward: 5'- TTATCCAACTCCCAACCCCT-3', reverse: 5'- TCCTGTTGCGAGTGCTTTAC-3') were designed according to the sequence amplified by the universal primer. The protocol of PCR action contains 12.8 μL of $ddH_2O$, 0.4 μL of forward

primer (10 μM), 0.4 μL of reverse primer (10 μM), 1 μL of DNA template, 3.2 μL of dNTP Mixture (2.5 mM each), 2 μL of 10×LA Taq Buffer II ($Mg^{2+}$ Plus) and 0.2 μL of TaKaRa LA Taq (5 U/μL). PCR reactions were performed under the following conditions: 4 minutes denaturation of 95 °C, followed by 35 cycles of 95 °C for 30 seconds, 59 °C for 30 seconds and 72 °C for 40 seconds, and final extension at 72 °C for 7 minutes. The PCR products were confirmed by the agarose gel electrophoresis.

**Activity assay of defensive enzymes**

One fungus-treated (or control-treated) termite and five nestmates were cultivated together for 5 d. There were 15 replicates of interactive cultivation for pathogenic fungus treatment and control treatment, respectively. Then, 15 nestmates from three replicates were pooled and crushed by liquid nitrogen and dissolved in a proportion of 1 mg of body weight to 10 μL of PBS. The homogenates were centrifuged at 10,000 × g for 15 min at 4 °C and the supernatant as samples of enzyme activity assay was used for analysis. We determine protein concentrations using bovine serum albumin as the standard[1]. The activities of superoxide dismutase (SOD, 5 replicates per treatment) and catalase (CAT, 5 replicates per treatment) were determined according to the protocols offered by manufacturer (Nanjing Jiancheng Bioengineering Institute, Jiangsu, China). To determine SOD activity, we used the systems of xanthine reacting with xanthine oxidase and measured their absorbance spectrophotometrically at 550 nm. One unit of SOD activity was determined as the number of enzyme required for 50% inhibition of the xanthine and xanthine oxidase system reaction in 1 mL enzyme extraction of 1 mg protein. SOD activity was expressed as U $mg^{-1}$ protein. To calculate CAT activity, we measured the amount of a faint yellow complex produced by surplus $H_2O_2$ reacting with ammonium molybdate spectrophotometrically at 405 nm. One unit of CAT activity was defined as the amount decomposing $H_2O_2$ per second per mg protein. CAT activity was also expressed as U $mg^{-1}$ protein.

**Expressions of immune genes**

One fungus-treated (or control-treated) termite and five nestmates were cultivated together for 5 d. The five nestmates were pooled and crushed in 1.5 mL centrifuge tube with liquid nitrogen, using sterilized disposable tissue grinding pestles. Total RNA of samples was extracted using TRIzol according to manufacturer protocol. The purity and concentration of the extracted RNA were determined by Thermo NANO DROP 2000 Spectrophotometer. Approximately 1μg RNA was treated by DNase-I, and then was converted to single-stranded cDNA using PrimeScript RT reagent Kit (perfect real time) (TaKaRa, Dalian, China). The cDNA products were then diluted with deionized water to 25-fold as template for the real-time PCR of *phenoloxidase* and 125-fold as templates for the real-time PCR of *transferrin*, *termicin* and *β-actin* (reference gene). The quantitative reaction was performed on My IQ™ 2 Two color Real-time PCR Detection System (Bio-Rad, USA). Reaction mixtures, containing 10 μL of SYBR Premix Ex Taq™ II (TaKaRa, Dalian, China), 0.4 μL of forward primer (10 μM), 0.4 μL of reserve primer (10 μM), and 2 μL of template cDNA were performed as the following reaction condition: 95 °C for 3 min, followed by 40 cycles of 95 °C for 10 s and 58 °C for 30 s.

According to the sequences of the other termites, primers were designed using Primer Premier 5. Based on the acquired sequences of *R. chinensis*, specific primers for real-time PCR were designed by Beacon Designer 7.7. The expressions of fungus-treated groups were calibrated by those of control-treated groups. The relative gene expressions were calculated by the method of $2^{-\Delta\Delta Ct}$[2]. The real-time PCR were performed on three biological replicates each containing three technical replicates.

**Referential sequences and primers used for PCR amplification of immune genes.**

| Gene Name | Accession Number for Referential Sequence | Source | Orientation | (5′→3′) Primer Sequence |
|---|---|---|---|---|
| *β-actin* | DQ206832.1 | *Reticulitermes* | Forward | CTCAGGTGATGGTGTCTC |
| | | *flavipes* | Reverse | CAGGTAGTCGGTCAAGTC |
| *phenoloxidase* | KF277251.1 | *Coptotermes* | Forward | TGACAACAGCACTAACAGCCC |
| | | *formosanus* | Reverse | TCATCACCACCTTGCACCC |
| *transferrin* | AF535146.1 | *Mastotermes* | Forward | TCCAAAATACCCCATCAAGAC |
| | | *darwiniensis* | Reverse | AAGTGACGCAGAACCTCACTG |
| *termicin* | ID FJ184569.1 | *Reticulitermes* | Forward | GGACTCTCTGTATCCTGCTCG |
| | | *chinese* | Reverse | AGACATGGGTGCTAAAAATGG |
| *defensin* | Unigene 11869 | *Odontoterms* | Forward | TCATTCTGGCTGCCCTGT |
| | | *formosanus* | Reverse | GGTCGGATATCTTCCTGTTGTG |

**Primers used for real-time PCR verification of immune genes.**

| Gene Name | Orientation | (5′→3′) Primer Sequence |
|---|---|---|
| *β-actin* | Forward | CTCAGGTGATGGTGTCTC |
| | Reverse | CAGGTAGTCGGTCAAGTC |
| *phenoloxidase* | Forward | AGTTCAGGAAGCGTGTAA |
| | Reverse | CATTGGCATCTGGTGTATC |
| *transferrin* | Forward | ATGCACGGCTTGCCTGTTAT |
| | Reverse | TGTGTGCGCTCTGTGAGCA |
| *termicin* | Forward | TCGTCTTTCTGGTCGTAGTG |
| | Reverse | CAGTGGTGATAGAGATGATA |
| *defensin* | Forward | GTCTGCTGAACACAACAAT |
| | Reverse | GGTCACAGGTTACTCGTT |

**Protein preparation**

Termite samples were ground into powder in liquid nitrogen and extracted with Lysis buffer (7 M Urea, 2 M Thiourea, 4% CHAPS, 40 mM Tris-HCl, pH 8.5) containing 1 mM PMSF and 2 mM EDTA (final concentration). After 5 min of vigorous vortex, 10 mM DTT (final concentration) was added to the samples. The suspension was sonicated at 200 W for 15 min and then centrifuged at 30 000×g for 15 min at 4 °C. The supernatant was mixed well with 5× volume of chilled acetone containing 10% (v/v) TCA and incubated at -20 °C overnight. After centrifugation at 4 °C, 30 000×g for 15 min, the supernatant was discarded. The precipitate was washed with chilled acetone three times. The pellet was air-dried and dissolved in Lysis buffer (7 M urea, 2 M thiourea, 4% NP40, 20 mM Tris-HCl, pH 8.0-8.5). The suspension was sonicated at 200 W for 15 min and centrifuged at 4 °C, 30 000×g for 15 min. The protein in the supernatant was transferred to another tube and reduced with 10 mM DTT (56 °C for 1 h). Cysteine residues blocked with 55 mM iodoacetamide (IAM) (darkroom temperature for 1 h). Protein was precipitated with chilled acetone at -20 °C for 2 h. After centrifugation at 4 °C, 30 000×g for 15 min, the supernatant was discarded, and the pellet was air-dried for 5 min, dissolved in 500 μL 0.5 M TEAB, and sonicated at 200 W for 15 min. Finally, the samples were centrifuged at 4 °C, 30 000×g for 15 min. The supernatant was transferred to a new tube and

determined by the protein-dye method of Bradford (1976) using bovine serum albumin as a quantitative standard[1]. The proteins in the supernatant were kept at -80°C for further analysis.

**iTRAQ Labeling and Strong Cation Exchange Choematography (SCX) Fractionation**

Total protein (100μg) was taken out of each sample solution and then the protein was digested with Trypsin (protein: trypsin ratio = 30: 1) at 37 °C for 16 hours. After trypsin digestion, peptides were dried by vacuum centrifugation. Peptides were reconstituted in 0.5 M TEAB and processed according to the manufacturer's protocol for 8-plex iTRAQ reagent (Applied Biosystems). Samples were labeled with the iTRAQ tags as follow: 114-, 116- and 118-iTRAQ tags for control replicate Control 1, Control 2 and Control 3; and 115-, 117- and 119-iTRAQ tags for fungus-treated replicate Fungus 1, Fungus 2 and Fungus 3, respectively. The peptides labeled with respective isobaric tags, incubated for 2 h and vacuum centrifuged to dryness. SCX chromatography was performed with a LC-20AB HPLC Pump system (Shimadzu, Japan). The iTRAQ-labeled peptide mixtures were reconstituted with 4 mL buffer A (25 mM NaH2PO4 in 25% ACN, pH 2.7) and loaded onto a 4.6×250 mm Ultremex SCX column containing 5 μm particles (Phenomenex). The peptides were eluted at a flow rate of 1 mL/min with a gradient of buffer A for 10 min, 5-60% buffer B (25 mM NaH2PO4, 1 M KCl in 25% ACN, pH 2.7) for 27 min, 60-100% buffer B for 1 min. The system was then maintained at 100% buffer B for 1 min before equilibrating with buffer A for 10 min prior to the next injection. Elution was monitored by measuring the absorbance at 214 nm, and fractions were collected every 1 min. The eluted peptides were pooled into 20 fractions, desalted with a Strata X C18 column (Phenomenex) and vacuum-dried.

**LC-ESI-MS/MS Analysis Based on Q EXACTIVE**

The peptides (5 μg) were taken up into 10μL 2% ACN, 0.1% trifluoroacetic acid (TFA) solvent and injected onto a 2 cm C18 trap column (inner diameter 200 μm) connected resolving 10 cm analytical C18 column (inner diameter 75 μm) on a Shimadzu LC-20AD nanoHPLC. Each sample was loaded onto the column at 8 μL /min for 4 min, then the 44 min gradient was run at 300 nl /min starting from 2 to 35% buffer B (98%ACN, 0.1%FA), followed by 2 min linear gradient to 80%, and maintenance at 80% buffer B for 4 min, and finally return to 5% in 1 min.

The peptides were subjected to nanoelectrospray ionization followed by tandem mass spectrometry (MS/MS) in an QEXACTIVE (Thermo Fisher Scientific, San Jose, CA) coupled online to the HPLC. Intact peptides were detected in the Orbitrap at a resolution of 70 000. Peptides were selected for MS/MS using high-energy collision dissociation (HCD) operating mode with a normalized collision energy setting of 27.0; ion fragments were detected in the Orbitrap at a resolution of 17500. A data-dependent procedure that alternated between one MS scan followed by 15 MS/MS scans was applied for the 15 most abundant precursor ions above a threshold ion count of 20000 in the MS survey scan with a following Dynamic Exclusion duration of 15 s. The electrospray voltage applied was 1.6 kV. Automatic gain control (AGC) was used to optimize the spectra generated by the Orbitrap. The AGC target for full MS was 3E6 and 1E5 for MS2. For MS scans, the m/z scan range was 350 to 2000 Da. For MS2 scans, the m/z scan range was 100 to 1800 Da.

**Proteomics Data Analysis**

Raw data files acquired from the Orbitrap were converted into MGF files using Proteome Discoverer 1.2 (PD 1.2, Thermo), [5600 msconverter] and the MGF file were searched. Proteins identification was performed by using Mascot 2.3.02 (Matrix Science, London, UK) against database containing 15860 sequences.

For protein identification, a mass tolerance of 20 Da (ppm) was permitted for intact peptide masses and 0.05 Da for fragmented ions, with allowance for one missed cleavages in the trypsin digests. Gln->pyro-Glu (N-term Q), Oxidation (M), Deamidated (NQ) as the potential variable modifications, and Carbamidomethyl (C), iTRAQ8plex (N-term), iTRAQ8plex (K) as fixed modifications. The charge states of peptides were set to +2 and +3. Specifically, an automatic decoy database search was performed in Mascot by choosing the decoy checkbox in which a random sequence of database is generated and tested for raw spectra as well as the real database. To reduce the probability of false peptide identification, only peptides with significance scores (≥20) at the 99% confidence interval by a Mascot probability analysis greater than "identity" were counted as identified. And each confident protein identification involves at least one unique peptide.

For protein quantization, it was required that a protein contains at least two unique peptides. The quantitative protein ratios were weighted and normalized by the median ratio in Mascot. Proteins with 1.2-fold change between fungus-treated and control samples and *p*-value of statistical evaluation less than 0.05 were determined as differentially abundant proteins.

**MRM validation of differentially expressed proteins from iTRAQ**

A spectral library of all proteins in samples MS/MS data was generated on TripleTOF5600 (AB SCIEX, Foster City, CA), searched using ProteinPilot (AB SCIEX, Foster City, CA) and imported into Skyline software[3] where a library was built. Unique peptides (7-30 amino acids length) without modifications and missed cleavages were selected for MRMs. We initially monitored four transitions per peptide to ensure specificity with the criteria that at least four y-ions had the same elution profile and were in the same ratios as the spectral library, and to see that predicted retention times were observed. A test pool of the six samples (Fungus: n = 3; Control: n = 3) was digested as described in iTRAQ and was performed preliminary SRM assays used to determine where these proteins were detected.

Samples were digested as described and spiked with 20 fmol of β-galactosidase for data normalization. MRM analyses were performed on QTRAP5500 mass spectrometer (AB SCIEX, Foster City, CA) equipped with Waters nano Acquity Ultra Performance LC system. The Mobile phase consisted of solvent A, 0.1% aqueous formic acid and solvent B, 98% acetonitrile with 0.1% formic acid. Peptides were separated on a Kinetex C18 column (150 × 3.0 mm, 2.6 μm) at 300 nL/min, and eluted with a gradient of 5-8% solvent B for 2 min, 8-30% solvent B for 90 min, and followed by 30-80% solvent B for 3 min. For the 5500 QTRAP mass spectrometer, spray voltage of 2100 V, nebulizer gas of 20 p.s.i. and a dwell time of 10 ms were used. Multiple MRM transitions were monitored using unit resolution in both Q1 and Q3 quadrupoles to maximize specificity. Each MRM transition had a minimum dwell time of 10ms. Data analysis was performed using Skyling[3]. At least one unique peptide per protein was used for quantification. The top three abundant transitions for each peptide were used for quantification unless interference from the matrix was observed. There are three replicates for all MRM analyses. For variation analysis, *t*-test was used to compare each peptide for significance. Spearman's correlation was used to analyze the correlation between log ratios of the quantitative data from MRM and log ratios of the quantitative data from iTRAQ for 14 target proteins.

## REFERENCES


1. Bradford, M. M. A rapid and sensitive method for the quantitation of microgram quantities of protein utilizing the principle of protein-dye binding. *Anal. Biochem.* **72**, 248-254 (1976).
2. Van Hiel, M. B. *et al.* Identification and validation of housekeeping genes in brains of the desert locust *Schistocerca gregaria* under different developmental conditions. *BMC Mol. Biol.* **10**, 56 (2009).
3. MacLean, B. *et al.* Skyline: an open source document editor for creating and analyzing targeted proteomics experiments. *Bioinformatics* **26**, 966-968 (2010).
